# The October 2014 United States Treasury bond flash crash and the contributory effect of mini flash crashes

**DOI:** 10.1371/journal.pone.0186688

**Published:** 2017-11-01

**Authors:** Zachary S. Levine, Scott A. Hale, Luciano Floridi

**Affiliations:** 1 Oxford Internet Institute, University of Oxford, Oxford, United Kingdom; 2 Alan Turing Institute, London, United Kingdom; Universidad Veracruzana, MEXICO

## Abstract

We investigate the causal uncertainty surrounding the flash crash in the U.S. Treasury bond market on October 15, 2014, and the unresolved concern that no clear link has been identified between the start of the flash crash at 9:33 and the opening of the U.S. equity market at 9:30. We consider the contributory effect of mini flash crashes in equity markets, and find that the number of equity mini flash crashes in the three-minute window between market open and the Treasury Flash Crash was 2.6 times larger than the number experienced in any other three-minute window in the prior ten weekdays. We argue that (a) this statistically significant finding suggests that mini flash crashes in equity markets both predicted and contributed to the October 2014 U.S. Treasury Bond Flash Crash, and (b) mini-flash crashes are important phenomena with negative externalities that deserve much greater scholarly attention.

## Introduction

Between 9:33 and 9:45, on October 15, 2014, the U.S. Treasury Bond market experienced a flash crash. During this twelve-minute period, the yield on the 10-year U.S. Treasury bond dropped and recovered an extraordinary 1.6%. There was, and still is, no clear cause of the sudden and severe Flash Crash. In July 2015, researchers from the U.S. Department of Treasury, Board of Governors of the Federal Reserve System, Federal Reserve Bank of New York, U.S. Securities and Exchange Commission and the US Commodity Futures Trading Commission published a comprehensive report on the day’s events which “does not reveal a single cause for the volatility seen on October 15,” and raises the particular concern that “no clear link has been identified between the event window [at 9:33 ET] and the open of the U.S. equity market at 9:30 ET” [1]. This article addresses the causal uncertainty surrounding October 2014 U.S. Treasury Bond Flash Crash, and suggests the presence of a link between the opening of the equity market at 9:30 to the start of the Flash Crash at 9:33 on October 15, 2014.

In the next section, we first contextualize and explain our hypothesis as to how an increase in the number of mini flash crashes in equity markets could have contributed to the October 2014 U.S. Treasury Bond Flash Crash. We then explain the data and methods employed to investigate this potential relationship before turning to describe findings from the investigation. Finally, we discuss the implications and limitations of our findings and conclude with broader implications and next steps.

## Background

In this section, we provide background and motivation for study of flash crashes, the October 2014 U.S. Treasury Bond Flash Crash, mini flash crashes, and the possible relationship between the October 2014 U.S. Treasury Bond Flash Crash and mini flash crashes. We also explain how an increase in the number of mini flash crashes in equity markets from 9:30 to 9:33 on October 15, 2014 could have contributed to the October 2014 U.S. Treasury Bond Flash Crash.

### Flash crashes

U.S. asset markets have experienced four other major flash crashes, in addition to the October 2014 U.S. Treasury Bond Flash Crash. The expression “flash crash” was introduced on May 6, 2010, when a slumping stock market abruptly declined 5%, before rebounding 6% within a period of ten minutes. On April 23, 2013, stock market gains of over 1% made throughout the day were swiftly erased, and then restored, over the course of just three minutes. On March 18, 2015, the U.S. dollar lost over 3% of its value in just under four minutes, and then regained all of its losses in the following three minutes. Finally, on August 24, 2015, the U.S. stock market lost over 5% in the five minutes after normal market trading opened. Asset markets outside the U.S. have experienced flash crashes as well.

Apart from their direct financial impact, flash crashes are a serious problem because they pose a threat to public trust in markets. Volatility and trust are inversely related [2], and “less trusting individuals are less likely to buy stock” [3]. Consequently, lower levels of trust are associated with reduced publicly-provided liquidity [4]. Plus, the effects of trust erosion compound if its cause is a repeated mechanism [5], like flash crashes. It is important to note that these problems spill over to markets beyond just those experiencing a flash crash because financial assets, financial markets, and their degrees of stability are interconnected [6]. In fact, thanks to recent advancements—such as public access to investment opportunities in foreign geographies and asset classes—and a boom in the popularity of pre-securitized cross-asset bundles (e.g., exchange traded funds) today’s markets are more interconnected than ever before [7]. Interconnectedness, while bringing about many benefits, could exacerbate the consequences of flash crashes.

The October 2014 U.S. Treasury Bond Flash Crash was an especially troubling flash crash. First, because of the market in which the flash crash took place. The U.S. Treasury bond market is considered to be the largest, safest, and most liquid asset market in the world, while U.S. public debt is more widely and globally held than any other security [1, 8]. In addition, interest rates on U.S. Treasury bonds are used as barometers for determining global economic health [9], and as pegs for many other interest rates, including American mortgage and student loan rates [10, 11]. The detrimental ripple effects of a flash crash in the U.S. Treasury Bond market could easily exceed those caused by flash crashes elsewhere. Second, the October 2014 U.S. Treasury Bond Flash Crash is history’s oldest major flash crash with such great causal uncertainty: strong evidence has been put forth to explain the earlier, major flash crashes on May 6, 2010 [12, 13] and April 23, 2013 [14]. It is critical that causal uncertainty surrounding this event is reduced, as uncertainty introduces another threat to public trust [15] and, consequently, publicly-provided liquidity. At the same time, causal uncertainty impedes action to prevent similar events from occurring in the future.

For these reasons, this article focuses on the causal uncertainty surrounding the October 2014 U.S. Treasury Bond Flash Crash, and in particular on the unresolved concern that “no clear link has been identified between the [start of the U.S. Treasury Bond Flash Crash at 9:33] and open of the U.S. equity market at 9:30 ET” [1].

### Mini flash crashes

In the aftermath of the May 6, 2010 Flash Crash, Nanex LLC (henceforth simply Nanex), a data analytics firm, investigated sub-second trade data from stock market feeds and discovered single-stock flash crashes that were taking place in millisecond time windows [16]. Nanex introduced the existence of these unanticipated extreme events to the world in a 2010 blog post, in which they named the events “mini flash crashes” [17]. A mini flash crash was characterized in Nanex’s blogpost as an uninterrupted price decline or incline on a single stock of at least 0.8% within a period of less than 1.5 seconds.

Unlike larger flash crashes, mini flash crashes are remarkably frequent. A seminal paper by Johnson et al. [18], co-authored with Eric Hunsader, Nanex’s founder, detected 18,520 mini flash crash events between 2006 and 2011. Still today, about a dozen mini flash crashes occur daily in the U.S. stock market [14]. In fact, many market participants have stopped tracking mini flash crashes because they occur “so frequently” [19].

Indeed, one reason why mini flash crashes have not received the public attention of larger flash crashes is because of their high frequency. In addition, they only directly affect single stocks and not whole markets; their price fluctuations are comparatively less severe than those of larger flash crashes; and their duration exists largely outside the frame of real-time human observation. Furthermore, there has not been a substantial amount of strong evidence for mini flash crashes producing negative externalities. More support for the negative externalities of mini flash crashes should motivate a greater amount of public attention and study of these phenomena in the future.

Still, some early literature on mini flash crashes does exist. The existing research can be broadly categorized into that which investigates mini flash crashes’ origins, properties, and consequences.

Johnson et al. [18, 20] suggested mini flash crashes usually originate from machine-generated decision making. Johnson et al. [18] pointed out that the 18,520 mini flash crashes they studied mostly took place in time periods too short for human action. Johnson et al. [20] expanded on the earlier paper and demonstrated that the number of mini flash crashes substantially increases as the threshold for the duration of a mini flash crash falls below human response times, reinforcing the view that non-human actors are involved. Dugast & Foucault [14] agreed: they claimed that machine-generated decision making increases the likelihood of price reversals, and propose that the frequency of mini flash crashes will increase as the cost of trading on fast-information shrinks, or as access to machine-generated decision making grows. In terms of contextual explanations, Golub et al. [21] suggested that mini flash crashes are the result of regulatory holes, like the legality and widespread use of intermarket sweep orders (which, according to their study, initiate greater than two-thirds of mini flash crashes) as well as the fact that Regulation National Market System fails to protect a market’s depth.

As for the events themselves, mini flash crashes and their properties were first analyzed by Nanex. Nanex tracked and visualized thousands of mini flash crashes from 2006 to 2011, and revealed that the size of trades executed during a mini flash crash varied considerably [17]. Golub & Keane [16] analyzed 15,731 mini flash crashes from 2006 to 2011 and discovered that most mini flash crashes occur at the beginning and end of the trading day; that there is no correlation between sector and time of a mini flash crash; and that two-thirds of all mini flash crashes happen on the New York Stock Exchange (NYSE) and NASDAQ. A recent study from Demos et al. [22] added that the distribution of mini flash crashes is not normal.

The consequences of mini flash crashes were first considered by Johnson et al. [20], who suggested that there “may indeed be a degree of causality between propagating cascades of UEEs [ultrafast extreme events] and global instability, despite huge differences in their respective timescales.” However, only Golub et al. [21] have quantifiably assessed the negative externalities of mini flash crashes. In a study of 5,140 crashes during four months in 2008 and 2010, Golub et al. [21] found that mini flash crashes increase transaction costs through the “detrimental impact [mini flash crashes have] on the Exchange Spread and NBBO [National Best Bid and Offer] Spread.” Specifically, they observed that the NBBO Spread and Exchange Spread increased an amazing 142% and 188% respectively, in the 60 seconds after a mini flash crash. In addition, Golub et al. [21] provided evidence to support the notion that mini flash crashes negatively affect market depth, as “volume at the NBBO decreases after the mini Flash Crashes.” According to their study, offers to buy stocks in the NBBO market declined by 19%, and offers to sell in the NBBO market declined 9%, after a mini flash crash. Also after a mini flash crash, offers to buy stock declined 38% on the exchange where the mini flash crash occurred. By offering strong evidence of mini flash crashes increasing transaction costs through widening the desired execution price between a market’s buyers and sellers, while at the same time decreasing the number of opportunities to buy and sell in a market, Golub et al. [21] corroborated and quantified the intuition that mini flash crashes do indeed harm market liquidity.

### The October 2014 U.S. Treasury Bond Flash Crash and mini flash crashes

The widespread use of portfolio diversification and other risk mitigation tactics, combined with recent advancements, have made markets more interconnected than ever before. Within this environment, today’s investors—primarily using machine-generated decision making—detect changes in status quo in a given market, and act at high speeds and with high volumes to lessen their exposure. Facing conditions that are perceived to be unstable and illiquid, investors search for markets that are more stable and liquid in which to invest in order to protect against risk [23]. We examine the possibility that, on October 15 2014, detections of instability and illiquidity in the stock market catalyzed quick and huge flights from that market into the Treasury bond market, since it is common, during unstable and illiquid times for investors to rush to the Treasury bond market as a “safe haven” [24]. Such a surge in demand for safe investments would result in a sudden and severe spike in prices for U.S. Treasury bonds as happened on October 15, 2014.

We examine whether there was a statistically significant increase in the number of mini flash crashes in the early minutes of trading on October 15, 2014. An increase in mini flash crashes could cause flight from the equity markets amongst investors using algorithm tools (as mini-flash crashes are generally only detectable with algorithms). Mini flash crashes are intrinsically both signals and actualizations of market instability. In addition, as Golub et al. [21] showed, mini flash crashes produce negative externalities of reducing market liquidity. If irregularities in the trends of mini flash crashes were detected in stock markets by those who were algorithmically monitoring mini flash crash patterns, it is reasonable to believe that they would have fled from stock markets and towards more stable and liquid U.S. Treasury bond markets, suddenly and severely inflating prices. Finding a significant increase in the number of mini flash crashes in the early minutes of trading on October 15, 2014 would help explain the origins of the October 2014 U.S. Treasury Bond Flash Crash and reduce the causal uncertainty surrounding the flash crash.

Of course, detecting an increase in the number of mini flash crashes in stock markets prior to the start of a subsequent larger flash crash would show that mini flash crashes could have helped predict a larger flash crash, like tremors before an earthquake. However, application of theory on market interconnectedness and extension of the idea that mini flash crashes hurt market liquidity make it plausible to state that an increase in the number mini flash crashes is not just predictive. Instead, a statistically significant increase in the number of mini flash crashes is likely to contribute to the larger flash crash. Thus, our hypothesis, supported in the following analysis, is that mini flash crashes in U.S. equity markets did indeed contribute to the U.S. Treasury Bond Flash Crash on October 15, 2014.

## Materials and methods

The objective of this study is to determine whether there was a statistically significant change between the number of mini flash crashes during the three-minute window before the start of the October 2014 U.S. Treasury Bond Flash Crash compared to other windows of the same duration. Specifically, we test the hypothesis that:

The number of mini flash crashes in the three-minute window from 9:30 to 9:33 on October 15, 2014, was significantly larger than the number of mini flash crashes in other three-minute window during the ten weekdays preceding October 15.

The analysis first identifies the number of mini flash crashes in the three-minute *test window* of 9:30 (inclusive) and 9:33 (exclusive) on October 15, 2014. This test window is between the market’s opening for regular trading, 9:30, and the beginning of the October 2014 U.S. Treasury Bond Flash Crash at 9:33 [1].

The number of mini flash crashes in the test window is compared to two control periods. The first comparison is to the number of mini flash crashes in the same time window of 9:30–9:33 during the ten weekdays preceding October 15. The second comparison is to the number of mini flash crashes during any three minute window during regular market hours (9:30am–4:00pm) during the same days.

Selection of the ten weekdays immediately prior to October 15 mitigates the effect of external trends such as macro events and seasonal variability on results. A control period of two weeks is also sufficient to determine baselines of data against which to compare the number of events in the test window.

Each block of three successive minutes in the control period is considered to be a window. A window begins at the 0th millisecond of every 0th second of every minute, and ends with the last trade prior to the 0th millisecond of every 0th second of every fourth consecutive minute. For the first portion of analysis, there are 10 three-minute control windows from 9:30 to 9:33 against which to compare results (one window each day). For the second portion of analysis, there are 388 windows per day, providing 3,880 three-minute control windows throughout all trading days. Because every minute is a part of three overlapping windows (except the first and last two windows of each day), every mini-flash crash is counted in multiple windows. This repeated counting is not an issue, since the objective of the analysis is to measure the number of mini flash crashes in the test window against the number of mini flash crashes in each window of similar duration during the control period.

The definition of a mini flash crash is adopted from the existing literature. Johnson et al. [18] expanded on the criteria of a mini flash crash introduced by Nanex [17] giving the following criteria: the stock price must tick down (or up) at least ten times before ticking up (or down); the price change has to exceed 0.8% of the initial price; and the entire event must occur within 1500 milliseconds. These same criteria were utilized by Golub et al. [16, 21] in their later studies of mini flash crashes.

Mini flash crashes that could have occurred within a larger price decline are not included in our analysis. As the literature defines a flash crash as concluding with an up (or down) tick, a decline (or spike) which meets all other criteria but fails to meet this last requirement is not considered a mini flash crash. Additionally, as the literature fails to elaborate further on the duration of a mini flash crash, we choose to mark the beginning of the 1500 milliseconds with the timestamp of the first down (or up) tick, and the end of the 1500 milliseconds with the timestamp of the lowest (or highest) price in a mini flash crash.

The data used in this paper was purchased from Nanex. In addition to researching mini flash crashes, Nanex, as mentioned above, is a leading provider of millisecond-timestamped U.S. stock market data. Johnson et al. [18, 20] and Golub et al. [16, 21] also used Nanex data for their analysis.

The raw data acquired from Nanex consisted of every trade, timestamped to the millisecond, which took place from October 1, 2014 to October 15, 2014 on the NYSE and NASDAQ, and NYSE MKT (formerly AMEX). We chose the NYSE and NASDAQ as exchanges for analysis because they are the world’s two largest stock exchanges; they together experience over 60% of daily U.S. equity trade volume; and experience more than two thirds of all mini flash crashes [16]. In addition, they are more closely monitored than any of the world’s other exchanges, and presumably investors are more likely to detect and quickly act upon instabilities in these exchanges.

There are four main limitations to this study. First, the data was acquired from Nanex, a third party. While Nanex is a leading provider of millisecond-timestamped stock market data and even provided the data used in earlier research, the data was not collected first-hand. Second, due to the interconnectedness of markets and opaque strategies of traders, it is impossible to show direct cause-and-effect. The tangled nature of financial markets results in this paper only being able to make a strong, supported argument of mini flash crashes playing a contributory role. Third, the argument that mini flash crashes played a contributory role could benefit in the future from supplementary analysis, such as parallel analysis of liquidity levels that was time-aligned with the increase in mini flash crashes. Showing a pattern of fleeting liquidity that matched chronologically with the increase in mini flash crashes would enhance the argument for mini flash crashes’ contributory effect. Finally, and most importantly, this study only considers mini flash crashes in U.S. equities on three exchanges. Such a limited view simply cannot provide the whole picture. Future analysis done in relation to the October 2014 U.S. Treasury Bond Flash Crash should be done on mini flash crashes in other U.S. markets, especially on mini flash crashes in derivatives markets (since derivative markets exhibit more cross-market interconnectedness than other markets), and on mini flash crashes on the other public stock exchanges. Nonetheless, searching for mini flash crashes on the NYSE and NASDAQ was the best place to start.

## Results

We take four steps in our analysis. First we calculate the number of mini flash crashes in the three-minute window between 9:30–9:33 on the day of the October 2014 U.S. Treasury Bond Flash Crash. In the three-minute window on October 15, 2014—henceforth referred to as the *test window*—we find thirteen mini flash crashes. These thirteen mini flash crashes took place across eleven unique equities.

**Fig 1:**
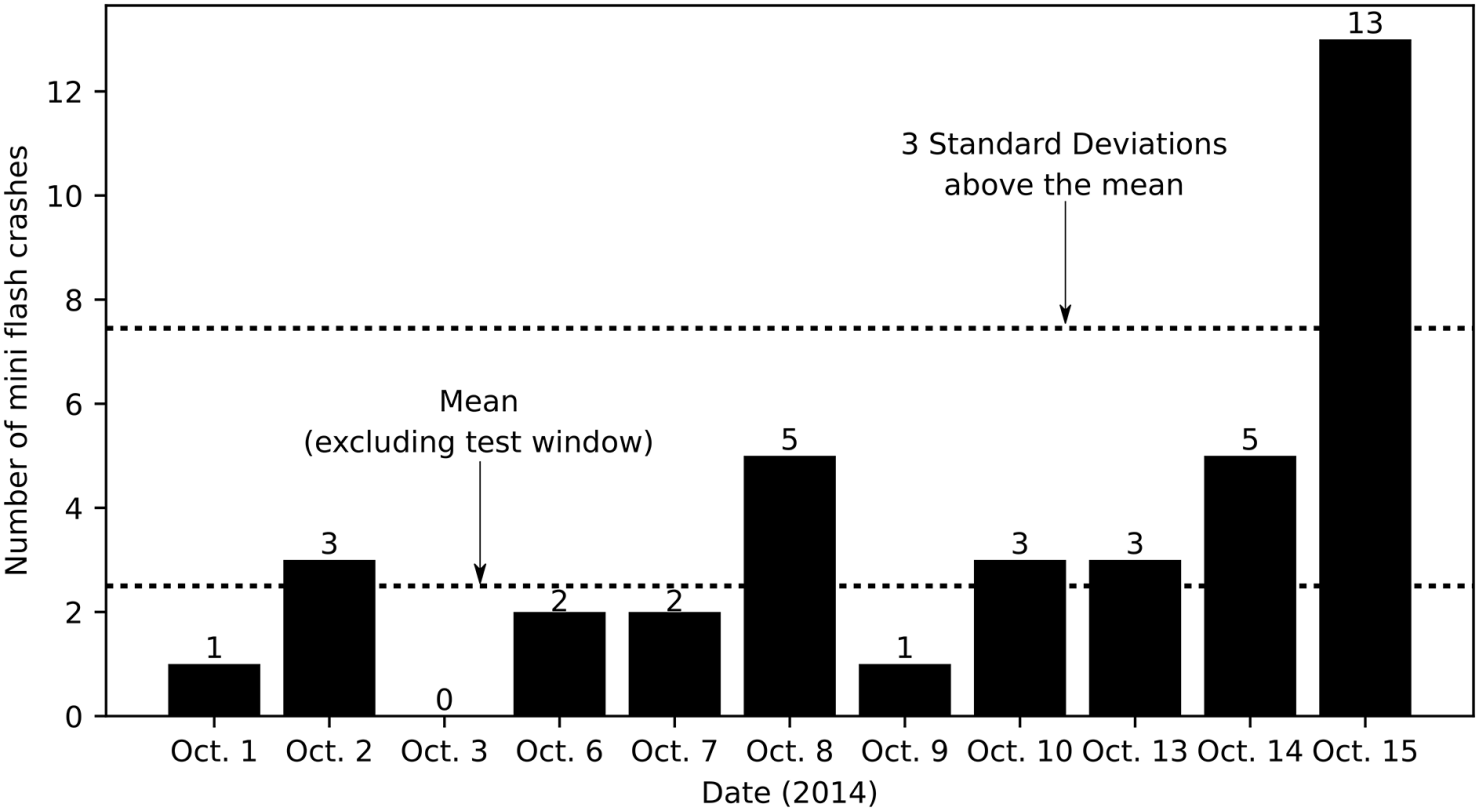
Number of mini flash crashes during Control Period 1 (9:30–9:33 windows). The number of mini flash crashes during the test window on Oct. 15 is 2.6 times greater than the greatest number of mini flash crashes experienced during any other 9:30–9:33 window.

Next, we calculate the number of mini flash crashes in *Control Period 1*, the three-minute windows between 9:30–9:33 each weekday during the 10 weekdays preceding October 15, 2014. In the three-minute windows between 9:30 and 9:33 during the control period, we detect a mean of 2.50 mini flash per day, with a standard deviation of 1.65 mini flash crashes. The number of mini flash crashes during the 9:30–9:33 window during the control period ranges from zero mini flash crashes on October 3, to five mini flash crashes on October 8 and October 14. The thirteen mini flash crashes experienced during the test window is 6.36 standard deviations above the 9:30–9:33 control mean, and is greater than 2.6 times the maximum number of mini flash crashes experienced on any day during the control period in the 9:30–9:33 window as shown in Fig 1.

**Fig 2:**
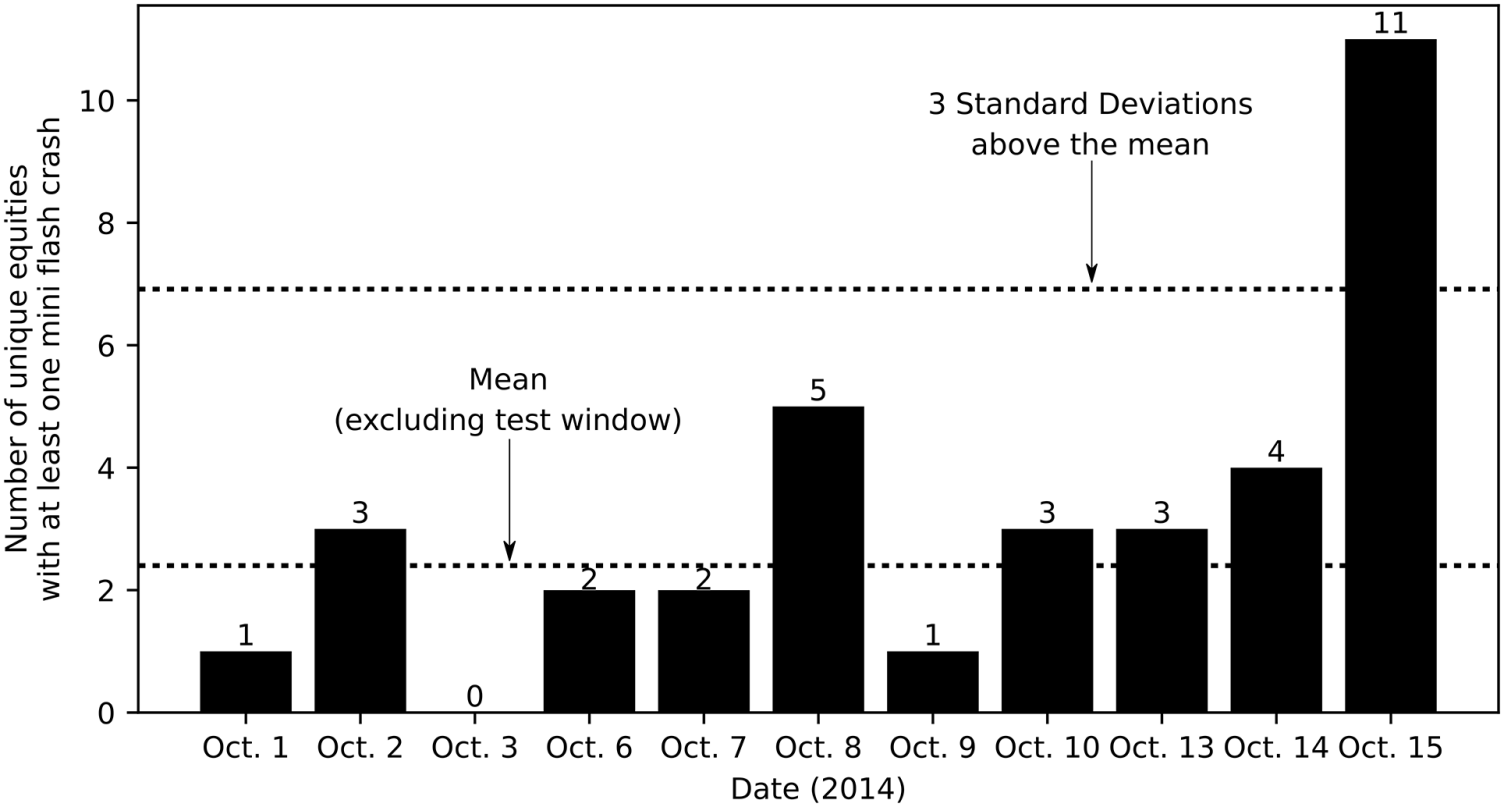
Number of unique equities that experienced mini flash crashes during Control Period 1 (9:30–9:33 windows). The number of unique equities that experienced mini flash crashes during the test window is 2.2 times greater than the greatest number of unique equities that experienced mini flash crashes during any other 9:30–9:33 window.

We consider the possibility of some equities experiencing a disproportionate share of the detected mini flash crashes. However, multiple mini flash crashes occurred on the same equity in Control Period 1 only once, on October 14, when one equity experienced two mini flash crashes. On average, 2.40 unique equities experienced a mini flash crash between 9:30–9:33 during the period, with a standard deviation of 1.51 unique equities. The number of unique equities that experienced a mini flash crash ranges from zero on October 3, to five on October 8. The eleven unique equities that experienced mini flash crashes during the test window is 5.71 standard deviations from the mean of Control Period 1, and 2.2 times the maximum number of unique equities that experienced mini flash crashes during the same period as shown in Fig 2.

We also consider the possibility that increased trading activity affected the absolute number of mini flash crashes (see Table 1). Indeed, the test window experienced more trades executed than any other 9:30–9:33 window during Control Period 1. However, despite the increase in the number of trades executed, the test window still experienced more mini flash crashes on a normalized scale than any other 9:30–9:33 window during Control Period 1. The test window experienced 1 mini flash crash per 56,183 trades, whereas on average during the control period, 1 mini flash crash was experienced per 186,407 trades. Regardless, it would be a mistake to over-emphasize the number of mini flash crashes per the number of total trades because detection of instability could beget greater quantities of trading. For instance, twelve of the thirteen mini flash crashes during the test window occurred in the sixty seconds between 9:30 and 9:31. This detection of instability could have certainly contributed to more trading activity during the remainder of the three-minute test window. Therefore, because the number of total trades could be affected by the number of mini flash crashes, mini flash crashes and trade activity cannot be viewed as independent events.

**Table 1:** Total trades executed and mini flash crashes during Control Period 1.

Date	Total trades	MFCs^†^	Trades per 1 MFC	Unique equities with MFCs
Oct. 1	354,394	1	354,394	1
Oct. 2	357,221	3	119,074	3
Oct. 3	364,482	0	n/a	0
Oct. 6	377,942	2	188,971	2
Oct. 7	368,060	2	184,030	2
Oct. 8	320,907	5	64,181	5
Oct. 9	354,617	1	354,617	1
Oct. 10	593,973	3	197,991	3
Oct. 13	376,676	3	125,559	3
Oct. 14	444,223	5	88,845	4
Oct. 15	730,379	13	56,183	11

^†^MFC: Mini flash crash.

The average number of trades per mini flash crash excluding the test window was 156,500.

An increase in the absolute number of mini flash crashes as shown in Fig 1 on its own suggests instability. In fact, it is reasonable to infer that machine-generated decision makers assess and act upon perceived market instability using this metric. Still, after accounting for the possibilities of some specific equities experiencing a disproportionate share of mini flash crashes, and variations in trading activity creating more opportunities for mini flash crashes to occur, the evidence continues to suggest that an abnormal level of instability could have been detected in the U.S. equity market during the test window on October 15, 2014. The number of mini flash crashes experienced during the test window relative to other 9:30–9:33 windows is abnormal because (a) the thirteen mini flash crashes during the test window is 2.6 times greater than the greatest number of mini flash crashes experienced during any other 9:30–9:33 window; (b) the eleven unique equities that experienced a mini flash crash during the test window is 2.2 times greater than the greatest number of unique equities which experienced a mini flash crash during any other 9:30–9:33 window; and (c) the frequency of mini flash crashes normalized by the number of trades executed is greater during the test window than it was during any other 9:30–9:33 window.

Our third step is to compare the number of mini flash crashes during the test window to the number of mini flash crashes in Control Period 2, which contains all three-minute window during throughout normal trading hours during the same 10 proceeding weekdays. While the literature suggests that mini flash crashes occur most frequently at the beginning and end of trading hours [21], we investigate all three-minute windows in the control period to check that the activity seen in the test window is truly anomalous from normal trading activity.

**Fig 3:**
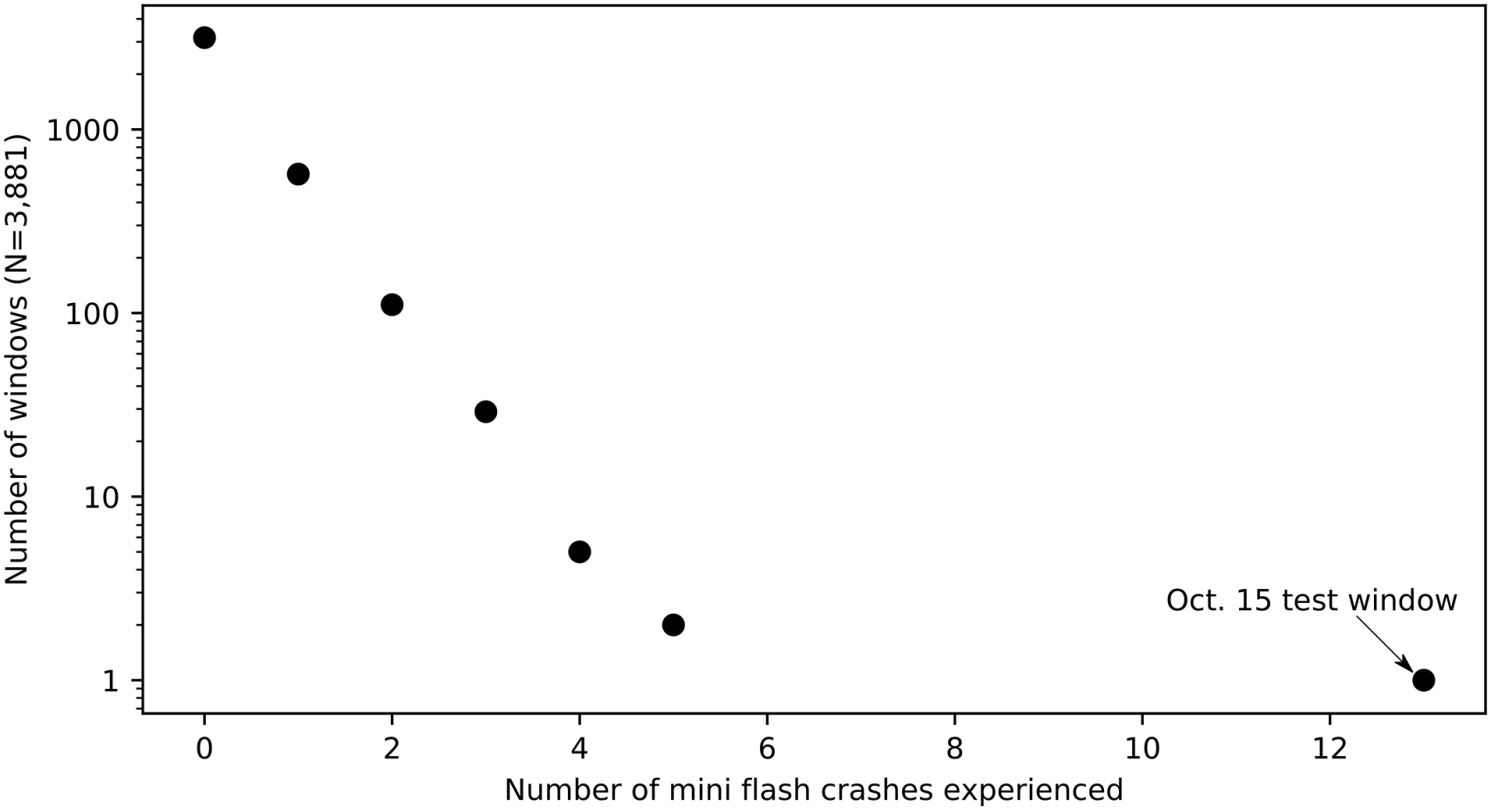
Distribution of number of mini flash crashes per three-minute window. The number of mini flash crashes during the test window on Oct. 15 is an extreme outlier. The thirteen mini flash crashes experienced during the test window are more than 2.6 times larger than the number of mini flash crashes experienced during any other three-minute window in Control Period 2.

There are 3,880 three minute windows during market hours during Control Period 2. 3,162 windows, or 81% of windows, experienced zero mini flash crashes. 517 windows experienced one mini flash crash; 111 windows experienced two mini flash crashes; 29 windows experienced three mini flash crashes; 5 windows experienced four mini flash crashes; and 2 windows experienced five mini flash crashes. There is not a single three-minute window during Control Period 2 that experienced more than five mini flash crashes, whereas the test window experienced thirteen mini flash crashes. Additionally, the thirteen mini flash crashes during the October 15 test window is 2.6 times the maximum number of mini flash crashes experienced on any day during any three-minute window throughout the control period as shown in Fig 3.

Our final step is to understand the statistical probability of observing the extreme result of thirteen mini flash crashes in a three-minute window. In order to quantify the anomalous nature of the test result, we calculate the probability (a p-value) from an exponential distribution fit to the mini flash crashes during Control Period 2 (that is, the distribution of mini flash crashes in all three-minute windows shown in Fig 3, fit to an exponential distribution). An exponential distribution is not a perfect fit for the data. However, an exponential distribution provides a more stringent and conservative test for statistical significance than either a normal distribution or a Poisson distribution. It is also theoretically more appropriate as Demos et al. [22] showed the distribution of mini flash crashes is not normal, and a Poisson distribution would falsely assume that mini flash crashes are independent events.

The best fit for an exponential function to the data (including the three-minute windows with zero mini flash crashes) was achieved with a function of *y* = 2624.8*e*^−1.497*x*^. Given this model, we calculated that the probability of observing 13 mini flashes crashes within one three-minute window was exceedingly small (*p* < 10^−8^) and thus highly significant. This p-value is far below the customary threshold of 0.05 and shows that the number of mini flash crashes on October 15 differed significantly from all other three-minute windows during the control period. It is also clear that the test result is an outlier relative to all 9:30–9:33 windows, even though there are not enough observations to fit to a distribution from which a p-value could be calculated.

The statistically significant increase in the number of mini flash crashes in the moments leading up to the 2014 U.S. Treasury Bond Flash Crash is consistent with the idea that mini flash crashes may have predicted and contributed to an ensuing larger flash crash. According to the causal possibility that we described in the Background section, it is likely that mini flash crashes played a contributory role in the October 2014 U.S. Treasury Bond Flash Crash. We also argue that this finding is the strongest evidence to date of a “clear link” between the start of the flash crash and the market’s opening.

## Discussion

The discovery of a statistically significant increase in the number of mini flash crashes between 9:30–9:33 on October 15, 2014 helps reduce causal uncertainty surrounding the October 2014 U.S. Treasury Bond Flash Crash. Specifically, the finding helps answer the concern that “no clear link has been identified between the [start of the U.S. Treasury Bond Flash Crash at 9:33] and open of the U.S. equity market at 9:30 ET” [1].

The immediate implication of reducing uncertainty surrounding the October 2014 U.S. Treasury Bond Flash Crash is that action can be taken to prevent similar events from occurring in the future in the U.S. Treasury bond market. Regulators can implement policies to monitor mini flash crashes proactively and, among other preemptive actions, limit mass liquidity flights from one market to the U.S. Treasury bond market during instances of heightened instability. At the same time, investors can take it upon themselves to be proactive about tracking mini flash crashes in equity markets, and to integrate technical safeguards to moderate cross-market flight to safety, when instances of abnormal instability arise. The long-term implication is that investors and the public at large can have more trust in the security and liquidity of the U.S. Treasury bond market. And government regulators and others overseeing the U.S. Treasury bond market can be more confident about minimizing concerns relating to uncertainty with this finding in hand. Both of these changes should help the U.S. Treasury bond market remain the world’s most stable and liquid market.

Additionally, we provide strong evidence of a negative externality of mini flash crashes. This should motivate greater attention to and study of the consequences of mini flash crashes, including the retroactive investigation into patterns of mini flash crashes prior to other flash crashes that are surrounded by great causal uncertainty, such as the Flash Crash of the U.S. Dollar on March 18, 2015 [25]. The general importance of reducing causal uncertainty surrounding other historic flash crashes is similar to the importance of reducing causal uncertainty surrounding the October 2014 U.S. Treasury Bond Flash Crash: causal uncertainty threatens to erode trust in markets and impedes action to prevent similar events from occurring in the future.

Finally, it seems important to renew interest in researching the origins of mini flash crashes. Broader efforts to identify the origins of mini flash crashes would supply more research to a critical area in need of better understanding. Furthermore, addressing the causes of mini flash crashes would also allow for actions to be taken to prevent mini flash crashes from occurring. Thwarting mini flash crashes would remove the impact mini flash crashes have on markets’ monetary value and liquidity (independent of the impact they have through suggested contributory effects), and remove mini flash crashes as contributing factors of larger flash crashes. Hopefully, this would then reduce the frequency of sudden, severe and consequential flash crashes.

Future analysis on mini flash crashes should consider markets for other assets and non-U.S. markets. The majority of research on mini flash crashes until this point has taken place in relation to U.S. equities, even though other markets around the world are similarly experiencing mini flash crashes. The lack of diversity in research in regard to asset class and geography is a limitation to the outstanding body of research on mini flash crashes at the moment.

## Conclusion

In this article we find a statistically significant increase in the number of mini flash crashes in equity markets in the moments leading up to the October 2014 U.S. Treasury Bond Flash Crash. Theory regarding the interconnectedness of markets and logic regarding the negative effect of mini flash crashes on liquidity quantified by Golub et al. [21], support the idea that an anomalous increase in the number of mini flash crashes would have contributed to the larger flash crash in addition to simply having preceded it. Thus we argue that mini flash crashes played a contributory role, and that the findings from the study help reduce causal uncertainty surrounding the October 2014 U.S. Treasury Bond Flash Crash.

A reduction in causal uncertainty lessens a threat to the erosion of public trust and liquidity in the world’s safest securities market. Uncertainty can have significant effects on trust, which is key to public participation in markets. Robust public participation, in turn, drives strong market liquidity, which is critical to an effective market functioning. By reducing causal uncertainty, this paper helps lessen a threat to trust erosion in, and deterioration of, publicly-provided liquidity in the U.S. Treasury bond market.

In addition, this paper could contribute in the long-term to reducing instability, another threat to erosion in public trust and publicly-provided liquidity in markets. First, by discovering a contributing factor to the October 2014 U.S. Treasury Bond Flash Crash, this paper lowers impediments to action by both regulators and investors to prevent similar events from occurring in the U.S. Treasury bond market in the future.

Second, this paper joins Golub et al. [21] as one of the few to investigate the negative externalities of mini flash crashes. The strong evidence of negative externalities presented in this paper could inspire retroactive investigation into patterns of mini flash crashes prior to other past flash crashes that are surrounded by great causal uncertainty. More importantly, it could also inspire study in and be part of a larger collection of evidence to support the predictive and contributory effects of mini flash crashes on larger flash crashes or on market crashes more broadly. In this case mini flash crashes could be added to the already diverse set of factors linked to market crashes such as economic freedom [26], transparency [27], and differences of opinion amongst investors [28]. This is the most significant potential contribution of this paper, as helping to reduce market crashes in the future would better preserve the monetary value and liquidity of U.S. markets, which are relied upon to bring about global and domestic welfare.
